# Comparative effectiveness of various intubation fixation devices for patients undergoing tracheal intubation in the ICU: A systematic review and network meta-analysis

**DOI:** 10.1016/j.ijnss.2025.12.009

**Published:** 2025-12-17

**Authors:** Zexi Huang, Nianqi Cui, Yanmin Zheng, Jianyan Yang, Yueli Ping, Ruiqin Sha, Yonggang Liu, Ying Tian

**Affiliations:** a School of Nursing, Kunming Medical University, Kunming, China; b The First Affiliated Hospital of Kunming Medical University, Kunming, China

**Keywords:** Catheter displacement, Endotracheal tube, Intensive Care Units, Intubation fixation devices, Network meta-analysis

## Abstract

**Objectives:**

This study aimed to compare and rank the efficiency of various devices used for intubation fixation in patients undergoing orotracheal intubation in the ICU.

**Methods:**

A comprehensive search of pertinent databases was conducted, encompassing all available records from their inception until March 2025. The databases included PubMed, Embase, Web of Science, the Cochrane Library, China Knowledge Resource Integrated Database (CNKI), Wanfang Databases, Chinese Biomedical Databases disc (CBM disc), and VIP Database. The review concentrated on randomized controlled trials (RCTs) that examined various intubation fixation devices for patients with tracheostomy in the ICU. A network meta-analysis was then performed to compare and rank the efficacy of these devices, with catheter displacement, facial pressure injuries, and patient-reported pain as the primary outcomes, by evaluating the odds ratios (*ORs*) and rank probabilities.

**Results:**

This network meta-analysis synthesized data from 16 RCTs assessing five different intubation fixation devices. No significant differences were found among devices regarding catheter displacement or facial pressure injuries. However, hybrid fixation (using adhesive tapes and straps) provided significantly better pain relief than dental pad fixation (*OR* = 16.27, 95 %*CI*: 3.56, 74.30) and had the highest ranking probability (78 %) for minimizing pain.

**Conclusions:**

While no single device was superior across all outcomes, hybrid fixation demonstrated a significant advantage in reducing patient pain. Future high-quality RCTs with standardized outcomes are needed to strengthen these findings and inform clinical guidelines.

## What is known?

• Endotracheal intubation is a crucial life-saving technique in ICUs, and effective catheter fixation is vital for safe mechanical ventilation.
• Poor orotracheal tube fixation increases the risk of complications, leading to ventilation problems and hypoxia, which complicate clinical management and raise medical resource usage.
• Although various fixation devices are available, their effectiveness, safety, and usability vary, necessitating further evidence-based research to identify the most effective solution.

## What is new?

• Compared with dental pad fixation, hybrid fixation can reduce patient pain.
• Nursing staff should be encouraged to implement hybrid fixation for awake patients who have undergone orotracheal intubation, as this approach may reduce patient pain and decrease the incidence of self-extubation.
• There were no statistically significant differences observed among the devices regarding catheter displacement or the incidence of facial pressure injuries in patients.

## 1 Introduction

Endotracheal intubation is a widely utilized method for establishing an artificial airway. It is frequently employed in emergency departments and ICUs to facilitate effective ventilation in critically ill patients [1]. Research indicates that nearly 70 % of ICU admissions involve patients who receive artificial airway treatments [2]. In clinical practice, orotracheal intubation is the most common method for establishing an artificial airway because it is fast, easy to perform, and highly successful. This method accounts for more than 96 % of all tracheal intubations [3]. Although effective in facilitating patient ventilation, orotracheal intubation is associated with certain limitations. Notably, complications such as catheter displacement [2], facial pressure injuries [4], and reduced patient tolerance [5] frequently occur due to insufficient catheter stabilization. Catheter displacement is one of the most prevalent complications encountered [6]. Numerous scholarly reviews have indicated that 60 % of unplanned extubation (UEX) incidents are attributable to inadequate catheter fixation techniques [7,8]. In addition, the improper fixation of tracheal catheters, which leads to displacement and dislodgement, is responsible for 9.7 %–43.7 % of these UEXs [9].

Currently, various methods for tracheal intubation fixation have been investigated both domestically and internationally. These methods include tracheal intubation fixators, bandage fixation, adhesive tape fixation, and dental pad fixation [10]. A comprehensive assessment revealed that each type of intubation fixation device has distinct pros and cons in clinical settings, with differences in their effectiveness in minimizing related complications [[11], [12], [13]]. Notably, compared with adhesive tape, the tracheal intubation fixator significantly reduces tube displacement and enhances patient tolerance [14]. A randomized controlled trial (RCT) further corroborated its superior stability during chest compressions relative to bandage fixation [15]. Alternating fixation with adhesive tape has been shown to optimize protection for the lingual mucosa and teeth [16]. Bandage fixation is particularly effective in lowering pressure ulcer risk scores and minimizing incidents of tube dislodgment and aspiration, offering greater precision than adhesive tape [12,13]. Although dental pad fixation is commonly employed in clinical practice, several international studies have indicated that it may lead to pressure injuries of the oral mucosa or damage to teeth [17,18]. In summary, the findings indicate significant variations in effectiveness and safety among different orotracheal intubation fixation devices. Furthermore, the absence of a systematic review or comprehensive meta-analysis of these devices contributes to the ongoing controversy regarding the optimal fixation device for patients undergoing orotracheal intubation in the ICU.

Network meta-analysis (NMA) is a methodological approach that facilitates the simultaneous evaluation of the effectiveness and safety of multiple interventions [19]. This method simultaneously assesses outcomes while integrating and contrasting both direct and indirect evidence. Consequently, to inform clinical decision-making, we conducted a systematic review and NMA to evaluate and rank the effectiveness of various fixation devices for patients undergoing orotracheal intubation in the ICU.

## 2 Methods

This NMA was conducted in accordance with the Preferred Reporting Items for Systematic Reviews and Meta-Analyses - NMA (PRISMA-NMA) statement [20]. This study is registered with the Prospective Register of Systematic Reviews (PROSPERO) (CRD42024567926).

### 2.1 Data sources and searches

Two researchers conducted a comprehensive electronic search across various databases, including PubMed, Embase, Web of Science, the Cochrane Library, China Knowledge Resource Integrated Database (CNKI), Wanfang Databases, Chinese Biomedical Databases disc (CBM disc), and VIP Database, to find relevant studies published until March 2025. The search strategy employed a combination of Medical Subject Headings (MeSH) terms and free-text terms, utilizing Boolean operators (“AND” and “OR”). The search terms included (“intubation, intratracheal” OR “intubation, indotracheal” OR “intubations, endotracheal” OR “endotracheal tube intubation”) AND (“respiration, artificial” OR “artificial respiration∗” OR “artificial ventilation” OR “artificial airway∗” OR “airway management” OR “airway interventions”) AND (“Intensive Care Units” OR “critical care” OR “ICU” OR “medical intensive care unit”) AND (“tube fastener” OR “tube-holder device” OR “adhesive tape” OR “cotton tape” OR “twill tape” OR “thomas tube holder”). A comprehensive search strategy is detailed in Appendix A (Table S1). Additionally, we reviewed the references of the included studies to identify any further relevant studies.

### 2.2 Inclusion and exclusion criteria

The inclusion criteria were established in accordance with the Population, Intervention, Comparison, Outcome, and Study Design (PICOS) framework [21]. 1) Population: all patients included in the study were adults (aged ≥18 years) who underwent orotracheal intubation. Patients had no neck or facial trauma, oral diseases, or a history of skin allergies. 2) Interventions: for patients undergoing orotracheal intubation, a variety of fixation devices are utilized, including tracheal intubation fixators (hard plastic clamp devices that secure the air tube catheter with a mechanical snap fastener), bandage fixations (non-adhesive nylon or cotton straps), adhesive tape fixations (medical adhesive tape using acrylic ester as an adhesive), dental pad fixations (silicone bite block secured with tape or bandages) and hybrid fixation (adhesive tapes and straps for fixation). Appendix A (Table S2) details each orotracheal intubation fixation device. 3) Comparisons: among the five types of fixation devices examined, the control group utilized one of the four types that the experimental group did not employ. 4) Outcomes: to thoroughly assess the efficacy of the endotracheal tube fixation device, three outcome measures were selected, encompassing its fundamental stability (catheter displacement), long-term tissue compatibility (facial pressure injuries), and patient tolerance (pain assessment). ① Catheter displacement [5]: monitoring of the distance from the hilar teeth for tracheal intubation. No displacement: the degree of the catheter tip from the incisors was completely unchanged. Mild displacement: the distance between the tip of the catheter and the scale of the incisor teeth is within 0.5 cm. Moderate displacement: the distance between the tip of the catheter and the scale of the incisors is 0.5–0.8 cm up and down, but it does not cause the catheter to fall off or slide down to one main bronchus. Severe displacement: the tube is dislodged, or the tube slides down and blocks one side of the bronchus. ② Facial pressure injuries [22] include the following: non-reddened white spots on pressure with intact skin; partial dermal loss with exposure of the dermis; total skin loss; total skin and tissue loss; and non-stageable total skin and tissue loss with the extent of damage masked; deep tissue damage persistent non-reddened white on pressure with a dark red, maroon or purple color. ③ Pain assessment [23]: Pain levels were evaluated utilizing a Visual Analogue Scale (VAS), categorized as follows: a score of 0 denotes the absence of pain; scores of 1–6 indicate tolerable pain; and scores ranging from 7 to 10 signify intolerable pain. 5) Study design: RCTs were included and published in Chinese or English.

Studies were excluded based on the following criteria: duplication of publications, lack of access to full-text articles, incomplete outcome data that hindered the calculation of effect sizes, sources not subjected to peer review, or the studies being protocols or ongoing trials.

### 2.3 Study selection and data extraction

All the search records were imported into EndNote X9 to facilitate the removal of duplicate studies. Subsequently, two researchers (Z. Huang and Y. Zheng) independently screened titles and abstracts to exclude studies that were not relevant to the topic. This was followed by a thorough review of the full texts to determine the final inclusion of studies. In cases of discrepancies, a third researcher (Y. Tian) was consulted to aid in the resolution process.

The primary data encompassed the following categories: 1) fundamental study details, including the year of publication, the first author, and the country of origin; 2) characteristics of the participants, such as sample size, gender distribution, age, primary diagnosis, disease severity score (Acute Physiology and Chronic Health Evaluation II, APACHE II), intervention duration and the duration of mechanical ventilation; 3) interventions, specifically the fixation devices utilized for endotracheal intubation; 4) outcome measures and their respective instruments, which included assessments of catheter displacement, facial pressure injury, and pain.

### 2.4 Quality appraisal

The revised Cochrane risk-of-bias (RoB 2) tool for randomized trials, designed explicitly for RCTs, was employed to assess the overall risk of bias for each included study [24]. It included five domains: bias arising from the randomization process, deviations from intended interventions (effect of assignment on intervention), missing outcome data, measurement of the outcome, and selection of reported results. This evaluation was conducted utilizing Microsoft Excel. For each domain, assessments were performed using a series of signaling questions, culminating in an overall risk-of-bias classification of “Low risk,” “Some concerns,” or “High risk” for each study outcome. The comprehensive assessment for a study was determined as follows: a “Low risk” rating was assigned if all domains were evaluated as low risk; a “Some concerns” rating was given if at least one domain indicated some concerns but no domain was rated as high risk; and a “High risk” rating was assigned if at least one domain was rated as high risk. Two researchers (Z. Huang and Y. Zheng) independently assessed the quality of the literature. In cases of significant disagreements, a discussion with a third researcher (N. Cui) was held to reach a decision. To ensure the preservation of network connectivity crucial for NMA, all eligible studies were incorporated into the primary analysis, irrespective of their risk of bias assessment. The potential influence of studies identified as having a high risk of bias on the outcomes will be examined through sensitivity analyses.

### 2.5 Data analyses

Data analysis was performed using R (version 3.6.2) and Stata (version 18.0). As the outcomes (catheter displacement, facial pressure injury, and pain) were dichotomous, they are presented as odds ratios (*ORs*) with 95 % confidence intervals (*CI*s). Specifically, the multi-level categorizations of these outcomes were consolidated into binary classifications as follows: catheter displacement was categorized as any displacement (encompassing mild, moderate, or severe) versus no displacement; facial pressure injury was classified as any injury (irrespective of stage) versus no injury; and pain was defined as any pain (VAS scores of 1–10) versus no pain (VAS score of 0). Considering that dental pad fixation is the predominant standard method for securing endotracheal tubes in the ICU [18,25], and recognizing that it is the most frequently utilized intervention among the studies included, with direct comparative evidence available for all other fixation devices, it has been designated as the traditional fixation method. This designation allows it to serve as the effective control group in the data analysis, thereby facilitating a comprehensive examination of both direct and circumstantial evidence.

A NMA was conducted under a random-effects model [26]. The network plot illustrated the available comparisons: node sizes were proportional to the sample sizes, and the thickness of the connecting lines represented the number of studies for each direct comparison. Heterogeneity was evaluated utilizing the *I*^2^ measure, which was classified as absent (0), low (25 %), moderate (50 %), or high (75 %) [27]. The consistency of the network was verified through both global and local tests, with a *P*-value greater than 0.05 indicating consistency [28]. Model convergence was confirmed through trajectory plots, density plots, and the potential scale reduction factor (PSRF ≈ 1). Results were presented in the form of a contingency table and probability-of-ranking plots, spanning a range of 0–100 %. Sensitivity analyses and comparative adjustment funnel plots were employed to assess the robustness of the results and to evaluate potential publication bias, respectively [29].

## 3 Results

### 3.1 Identification of relevant studies

Initially, a total of 16,917 studies were identified. Following the removal of duplicate entries, 6,909 articles were excluded. A subsequent evaluation of titles and abstracts resulted in the exclusion of an additional 9,671 articles. A comprehensive full-text review led to the exclusion of 322 articles. Through citation searching, one relevant publication was identified. Ultimately, only 16 RCTs, comprising 15 two-arm studies and one three-arm study, met the eligibility criteria and were included in the analysis [5,12,13,[30], [31], [32], [33], [34], [35], [36], [37], [38], [39], [40], [41], [42]]. A schematic flowchart illustrating the selection process of the included studies is presented in Appendix B (Fig. S1).

### 3.2 Characteristics of the included studies

The study included a total of 1,876 participants from research conducted in China, the United States, Singapore, and Turkey. The sample sizes varied from 18 to 153 participants, and the intervention durations ranged from 4 to 27 months. Five intubation fixation devices were utilized: tracheal intubation fixators (*n* = 6) [13,30,[33], [34], [35], [36]], dental pad fixation (*n* = 8) [34,[36], [37], [38], [39], [40], [41], [42]], adhesive tape fixation (*n* = 8) [5,12,[30], [31], [32], [33],^37^,^40^], bandage fixation (*n* = 8) [5,12,13,31,32,35,39,42], and hybrid fixation (adhesive tapes and straps for fixation) (*n* = 3) [33,38,41]. The primary characteristics of the included studies are presented in Appendix A (Table S3).

### 3.3 Risk of bias assessment

The concept of the “randomization process” was mentioned in 13 of the studies reviewed; however, only 15.4 % of these studies provided a detailed description of allocation concealment. Among the biases related to deviations from intended interventions, three studies explicitly reported on participant implementation and personnel blinding. The remaining studies strictly adhered to the intended intervention allocation without deviations. Regarding missing outcome data, 14 studies successfully collected all necessary data. Outcome measurement bias was evaluated in 13 studies, all of which were determined to have a low risk of bias. The risk of bias was also low with respect to the selective reporting of results. The overall bias assessment indicated that 13 studies were at possible risk [5,12,13,[31], [32], [33], [34], [35], [36], [37], [38],^40^,^42^], one was at low risk [30], and two were at high risk [39,41]. As pre-specified in the methods, all studies were retained for analysis. The risk of bias assessment for each included study is presented in Appendix B (Fig. S2).

### 3.4 Network meta-analysis results

#### 3.4.1 Catheter displacement

**Fig. 1:**
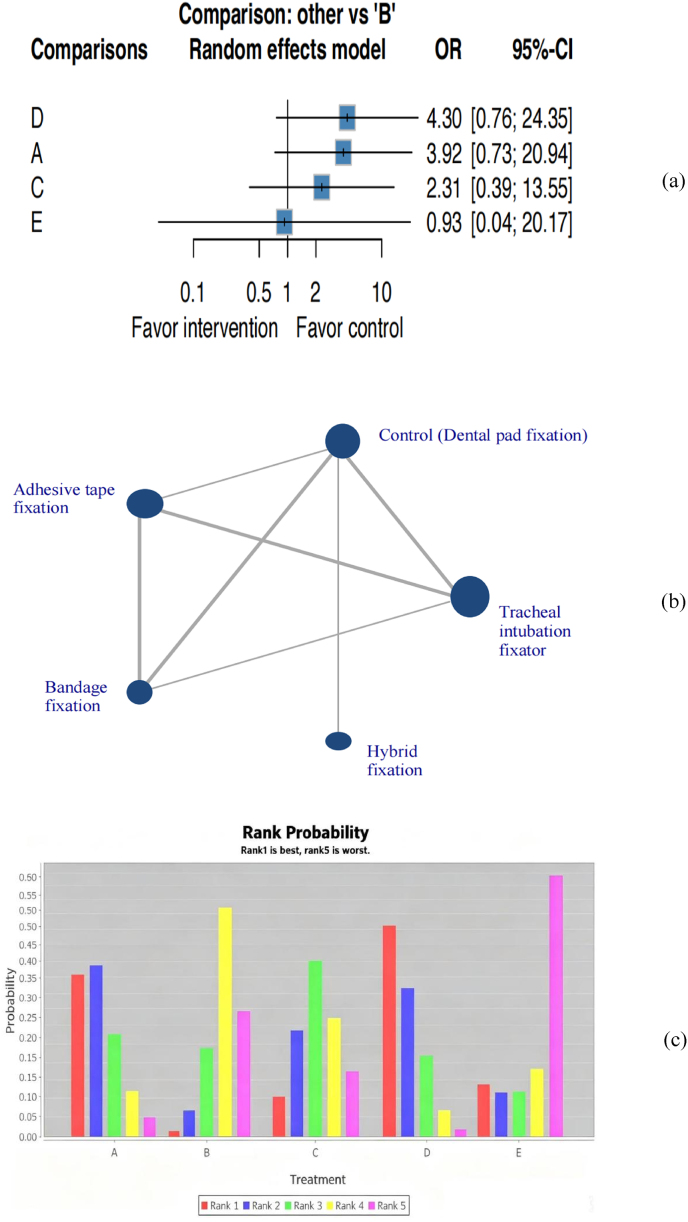
Direct paired random effects meta-analysis results (a), network graph (b), and probability-of-ranking plot (c) for catheter displacement outcomes. A = Tracheal intubation fixator; B = Control (Dental pad fixation); C = Adhesive tape fixation; D = Bandage fixation; E = Hybrid fixation.

Ultimately, 11 RCTs examined the impact of five distinct devices used for intubation fixation on catheter displacement in ICU patients who underwent orotracheal intubation [5,12,30,[33], [34], [35], [36], [37], [38], [39],^42^]. Direct paired random effects meta-analysis indicated no statistically significant differences in the effectiveness of the four fixation devices in reducing catheter displacement compared with traditional fixation devices (dental pad fixation) (Fig. 1a). Fig. 1b illustrates the network graph, which depicts the relationships among the devices. The PSRF values, evaluated using the Brooks–Gelman–Rubin method, approached 1, indicating that the statistical results were reliable (Appendix B, Fig. S3). Based on the interval estimation of both direct and indirect comparisons, no intubation fixation device exhibited statistically significant superiority over the other devices. A contingency table illustrating the comparison of various fixation devices is presented in Appendix B (Fig. S4). The results of the probability-of-ranking plot indicate the relative effectiveness of various fixation devices in preventing orotracheal catheter displacement. The order of effectiveness was as follows: bandage fixation, tracheal intubation fixator, adhesive tape fixation, dental pad fixation, and hybrid fixation (Fig. 1c). The consistency of the catheter displacement indicators was assessed using the node-splitting method. Node analysis revealed a significant inconsistency between adhesive tape fixation and bandage fixation (*P* = 0.04), whereas no inconsistencies were detected among the other interventions in pairwise comparisons (*P* > 0.05). Overall, the consistency was considered satisfactory (Appendix A, Table S4).

#### 3.4.2 Facial pressure injuries

**Fig. 2:**
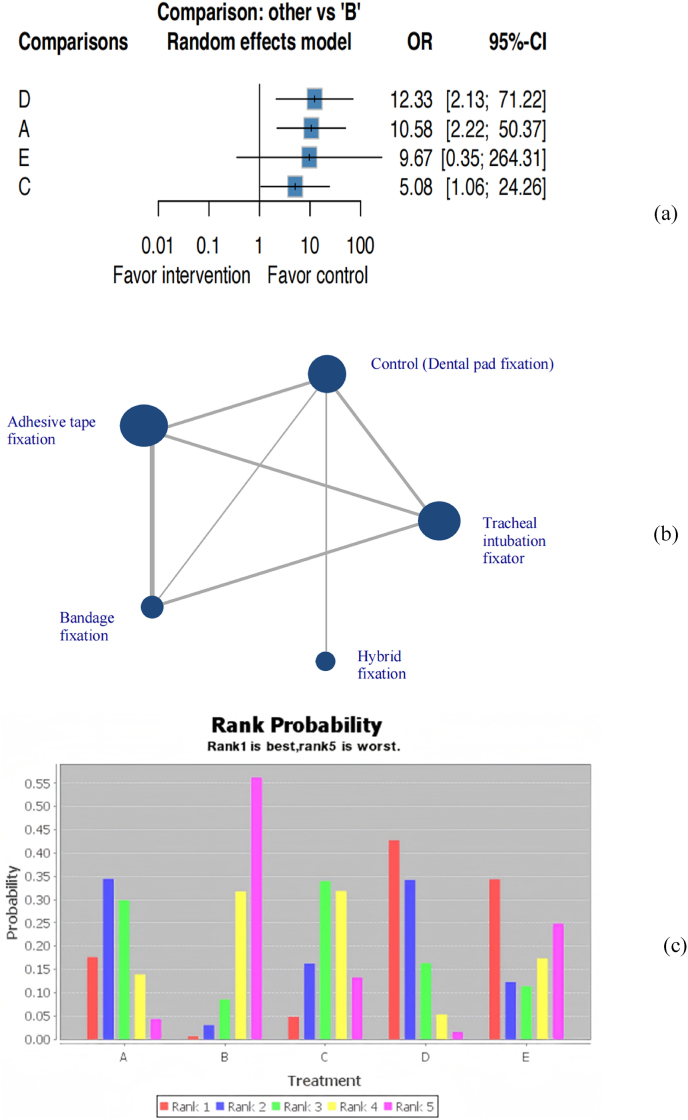
Direct paired random effects meta-analysis results (a), network graph (b), and probability-of-ranking plot (c) for facial pressure injury outcomes. A = Tracheal intubation fixator; B = Control (Dental pad fixation); C = Adhesive tape fixation; D = Bandage fixation; E = Hybrid fixation.

A comprehensive evaluation was conducted on 13 RCTs concerning facial pressure injuries in patients who underwent orotracheal intubation [5,13,[30], [31], [32], [33], [34], [35], [36], [37], [38],^40^,^42^]. In conclusion, the direct paired random effects meta-analysis demonstrated that, compared with traditional fixation devices, the use of a tracheal intubation fixator (*OR* = 10.58; 95 %*CI:* 2.22, 50.37) and bandage fixation (*OR* = 12.33; 95 %*CI*: 2.13, 71.22) significantly reduced the incidence of facial pressure injuries in patients undergoing orotracheal intubation in ICUs (Fig. 2a). Fig. 2b shows the network graph of the device. The model exhibited satisfactory convergence throughout the iterative calculations. The PSRF values, evaluated using the Brooks–Gelman–Rubin method, approached 1, indicating that the statistical results were reliable (Appendix B, Fig. S5). Based on the interval estimation of direct and indirect comparisons (Appendix B, Fig. S6), none of the intubation fixation devices demonstrated significant superiority over the other devices. The hierarchical arrangement of the devices is depicted in Fig. 2c. The probability-of-ranking plot indicates that the three most effective fixation devices for preventing facial pressure injuries in patients undergoing orotracheal intubation are, in descending order of efficacy, bandage fixation, tracheal intubation fixator, and adhesive tape fixation. Consistency was assessed using the node splitting method. The analysis revealed a significant inconsistency between adhesive tape fixation and bandage fixation (*P* = 0.04). Conversely, no discrepancies were detected among the other interventions in pairwise comparisons (*P* > 0.05). Overall, the consistency of the results was considered satisfactory (Appendix A, Table S5).

#### 3.4.3 Pain assessment

**Fig. 3:**
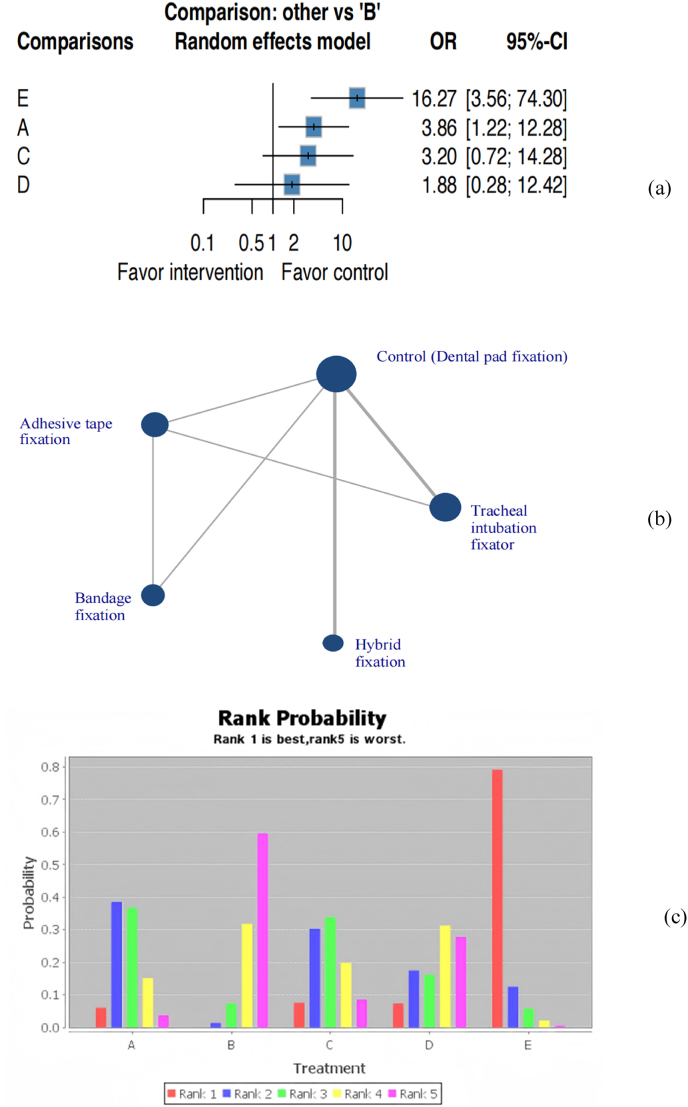
Direct paired random effects meta-analysis results (a), network graph (b), and probability-of-ranking plot (c) for pain outcomes. A = Tracheal intubation fixator; B = Control (Dental pad fixation); C = Adhesive tape fixation; D = Bandage fixation; E = Hybrid fixation.

A comprehensive analysis was conducted on eight RCTs and five distinct types of fixation devices to evaluate their impact on the pain of intubated patients [5,33,34,[36], [37], [38],^41^,^42^]. Overall, the use of a tracheal intubation fixator (*OR* = 3.86; 95 %*CI*: 1.22, 12.28) or hybrid fixation (*OR* = 16.27; 95 %*CI*: 3.56, 74.30) had a statistically significant positive effect on alleviating patient pain, as illustrated in Fig. 3a. Fig. 3b displays the network graph of the devices. The model exhibited satisfactory convergence throughout the iterative calculations. The PSRF values, evaluated using the Brooks–Gelman–Rubin method, approached 1, indicating that the statistical results were reliable (Appendix B, Fig. S7). Based on the interval estimation of direct and indirect comparisons (Appendix B, Fig. S8), the adoption of hybrid fixation methods is more effective than traditional fixation techniques. The probability-of-ranking plot demonstrated that, in terms of efficacy for alleviating pain in patients undergoing orotracheal intubation, the three most effective fixation devices were, in descending order, hybrid fixation, tracheal intubation fixator, and adhesive tape fixation (Fig. 3c). The consistency test was performed utilizing the node splitting method, and subsequent node analysis indicated that the outcomes demonstrated consistency between direct and indirect results from pairwise comparisons. The differences observed were not statistically significant (*P* > 0.05). These findings from the consistency test suggest that the evidence is stable and reliable (Appendix A, Table S6).

### 3.5 Heterogeneity test

The results of the heterogeneity test indicated a lower degree of variation in pain assessment but greater variability in catheter displacement and facial pressure injury across different patient proportions. Given the substantial number of devices analyzed, the extensive time frame of the studies, and the potential for significant clinical heterogeneity, we opted to employ a random-effects model to increase the generalizability of our findings (Appendix A, Table S7).

### 3.6 Sensitivity analysis

A sensitivity analysis was conducted on catheter displacement and facial pressure injury indicators. The magnitude of the combined effects did not exhibit significant changes upon the sequential exclusion of individual studies, indicating the stability of the data results. In the pain assessment sensitivity plot analysis, the combined effect size of one study fell outside the confidence interval for the overall effect size. However, the combined effect size remained essentially unchanged after excluding each survey individually, suggesting the stability of the data results. Notably, the exclusion of two high-risk studies did not significantly affect the statistical significance or alter the direction of the primary findings (Appendix B, Figs. S9–S11).

### 3.7 Publication bias

Upon examining the publication bias results related to catheter displacement, several data points clearly coincided with the red vertical line on the funnel plot. The data distribution within the funnel plot predominantly exhibited symmetry around this line. However, it is crucial to acknowledge that two data points fall outside the funnel boundaries, which may indicate the presence of publication bias. Notably, one point is situated at the bottom of the funnel, which may suggest a small sample effect and further imply potential publication bias. In the analysis of publication bias concerning facial pressure injury, three data points were located outside the funnel plot, and the distribution in the lower panel was asymmetrical, further suggesting the presence of publication bias. These observations may be attributed to the poor quality of the included studies and the small sample sizes (Appendix B, Figs. S12–13).

## 4 Discussion

### 4.1 Summary and interpretation of findings

Fixation is essential in orotracheal intubation procedures; however, it is important to note that both direct comparative analyses and NMA conducted in this review demonstrated no statistically significant differences in the effectiveness of various fixation devices in minimizing catheter displacement. The rank probability of effectiveness indicates that, in instances of catheter displacement, bandage fixation is most likely to achieve a rank of 1 among the five different fixation devices. This finding aligns with those reported in a prior systematic review [5,12,13]. The underlying mechanisms can be attributed to several factors. bandage fixation results in greater toughness and durability than adhesive tape does, maintaining its effectiveness in securing fixation even when exposed to facial or oral secretions. The mechanism of bandage fixation predominantly depends on the tension created by the frame and the friction between the knot and the catheter to maintain the position of the catheter. Although the band may deform when moved, its straightforward design allows nursing staff to easily adjust its tightness, lowering the risk of catheter displacement [43].

Compared with tracheal intubation fixators, bandage fixation can effectively reduce facial pressure injuries in patients undergoing orotracheal intubation in the ICU; however, broad *CI*s could affect the reliability of our estimates concerning the effectiveness of these interventions. However, the NMA revealed no statistically significant differences among the devices in terms of the incidence of facial pressure injuries in patients. These inconsistent results may be due to different methods of data pooling. For pairwise meta-analysis, data were directly pooled and compared, and the mutual influence among various interventions was not considered. For the NMA, the data were pooled and compared after adjustment. While the outcomes of the NMA generally do not exhibit statistically significant differences, the probabilistic ordering of results remains informative [44]. The probability-of-ranking plots indicated that banded fixation presented the highest likelihood of being ranked first among the five different fixation devices for patients with facial pressure injuries. This finding aligns with previous systematic reviews [39,45,46], which similarly identified banded fixation as the most likely optimal fixation device for enhancing the recovery of facial pressure injuries. Genc and Yildiz also reported that bandage fixation has a positive effect on mitigating facial pressure injuries in patients [13].

A total of eight RCTs comparing the pain of awake patients were included in this review. The probability-of-ranking plot of effectiveness suggested that hybrid fixation is most likely to rank highest in terms of alleviating patient pain, with a probability of 78 %. This finding is consistent with that reported in a previous systematic review [38,41]. Patients exhibit low tolerance for orotracheal intubation and frequently experience irritation when conscious, increasing the risk of accidental tracheal extubation. Studies have indicated that the incidence of UEX ranges from 10.8 % to 22.5 %, with self-extubation accounting for 87.5 %–96.0 % of these cases [47,48]. The evidence suggests that removing dental pads and implementing hybrid fixation in conscious patients effectively reduces oral discomfort [41]. Improved patient comfort is associated with fewer intentional extubations and a notable decrease in sedation requirements. Several factors may contribute to this positive effect. Patients who undergo orotracheal intubation frequently present with critical conditions characterized by physiological disorders, diminished body resistance, and decreased pain tolerance. The prolonged use of dental pads, which is necessary due to the patient's extended passive mouth opening, is likely to induce temporomandibular joint pain and soreness, which may subsequently lead to headaches. Upon removal of the dental pads, there was a notable alleviation of soreness and pain in the mandibular joints, as well as a reduction in headaches associated with their use. Furthermore, the elimination of friction between the pads and the oral mucosa and gingiva during placement significantly reduced the patient's oral discomfort. Moreover, most bandage fixation materials, usually made from cotton or fabric, can absorb the patient's oral secretions. This absorbency reduces the frequency of tape replacement, thereby reducing patient discomfort.

The application of the VAS for evaluating discomfort associated with fixation devices in this study presents certain limitations. In 50 % of the RCTs included in this analysis, the VAS score was designated as an indicator of comfort. While we adhered to its original definition when reporting results, it is crucial to clarify that the “pain” measured by the VAS pertains specifically to discomfort directly linked to the fixation device (e.g., skin irritation, adhesive tension) rather than pain in a broader context. Future research should prioritize the development or adoption of more targeted assessment tools. This could involve integrating the VAS with behavioral scales, such as the Richmond Agitation-Sedation Scale (RASS) [49], to enhance the validity of the assessment by incorporating both behavioral observations and physiological indicators.

This study demonstrated that various fixation devices exhibit differential performance across key outcome measures. According to existing evidence, the selection of the most appropriate fixation device should be closely tailored to the patient's specific condition and clinical requirements. For example, in agitated patients or those prone to biting the tube, dental pad fixation devices can effectively prevent the tracheal tube from being occluded or inadvertently displaced [50]. Conversely, for patients with delicate facial skin or excessive secretions, bandage fixation, which utilizes moisture-absorbing materials such as cotton/nylon blends and features adjustable tension properties, may offer enhanced potential in minimizing skin friction injuries [51]. Adhesive tape fixation provides a practical and suitable solution for emergencies; however, it poses a heightened risk of skin damage in patients with fragile skin or excessive perspiration due to its adhesive properties. Conversely, fixation devices offer robust support; however, their design necessitates positioning between the upper and lower teeth, which may potentially exacerbate oral discomfort in conscious patients [10]. Hybrid fixation effectively balances stability with patient comfort [52], making them particularly advantageous for scenarios that necessitate prolonged intubation, where both safety and tolerability are of paramount importance.

### 4.2 Strengths and limitations of this study

Our NMA offers several advantages. 1) By employing NMA, we conducted a comprehensive evaluation of the effects of five common clinical fixation devices on the stabilization of patients who underwent orotracheal intubation in the ICU. Furthermore, we employed probability-of-ranking plots to elucidate the differences among the interventions and to distinguish their relative effectiveness. 2) Given the limited number of RCTs concerning fixation devices for patients undergoing orotracheal intubation both domestically and internationally, we conducted an extensive database search to summarize and analyse the existing evidence.

This NMA is subject to several significant limitations. 1) The limited number of available RCTs, particularly those directly comparing catheter displacement (only two RCTs), constrains the robustness of our conclusions and precludes meaningful subgroup analyses. 2) Despite our rigorous standardization of intervention categories utilizing predefined functional classification, inherent heterogeneity in material composition and application techniques persists across devices with identical descriptive labels. This variation mirrors real-world clinical practice but adds analytical complexity. Residual heterogeneity arising from unmeasured material or technical differences may broaden *CI*s and obscure subtle distinctions in efficacy between specific device subtypes. 3) Although our comprehensive search strategy included major Chinese and English databases, 75 % of the included RCTs were conducted in China. This geographical concentration may limit the generalizability of our findings to other healthcare settings. Future multiregional RCTs are needed to enhance the external validity of the evidence.

## 5 Conclusions

This study conducted a comprehensive comparison of the effectiveness of five distinct orotracheal intubation devices. The results indicated no statistically significant differences among the devices concerning catheter displacement or the incidence of facial pressure injuries in patients. However, in comparison to traditional fixation devices, the hybrid fixation method has emerged as the sole treatment approach capable of effectively alleviating patient pain. The current literature on the efficacy of various intubation fixation devices for ICU patients undergoing orotracheal intubation is sparse. Consequently, there is an urgent need for further research, particularly high-quality RCTs utilizing standardized outcome measures, to validate these findings.

## Data availability statement

The datasets generated during and/or analyzed during the current study are available from the corresponding author upon reasonable request.

## CRediT authorship contribution statement

**Zexi Huang:** Conceptualization, Methodology, Software, Data curation, Writing - original draft, Writing - review & editing, Project administration. **Nianqi Cui:** Conceptualization, Methodology, Validation, Formal analysis, Funding acquisition, Writing - review & editing, Supervision, Project administration. **Yanmin Zheng:** Conceptualization, Methodology, Software, Data curation, Writing - original draft, Writing - review & editing, Project administration. **Jianyan Yang:** Methodology, Data curation, Writing - original draft, Writing - review & editing, Project administration. **Yueli Ping:** Conceptualization, Data curation, Writing - original draft, Writing - review & editing, Project administration. **Ruiqin Sha:** Conceptualization, Data curation, Writing - original draft, Writing - review & editing, Project administration. **Yonggang Liu:** Conceptualization, Methodology, Validation, Formal analysis, Investigation, Resources, Writing - review & editing. **Ying Tian:** Conceptualization, Methodology, Validation, Formal analysis, Funding acquisition, Writing - review & editing, Supervision, Project administration.

## Funding

This research was funded by the Science Research Foundation of the Chinese 10.13039/100028824Nursing Association [Grant No. ZHKY202304]. The funder had no role in the design and conduct of the study; collection, management, analysis, and interpretation of the data; preparation, review, or approval of the manuscript; and decision to submit the manuscript for publication.

## Declaration of competing interest

The authors have declared no conflict of interest.
