# Supplementary material for: Comparative effectiveness of various intubation fixation devices for patients undergoing tracheal intubation in the ICU: A systematic review and network meta-analysis
Source: Int J Nurs Sci. 2025 Dec 17;13(3):340–7. doi: 10.1016/j.ijnss.2025.12.009 (PMC13245521; doi:10.1016/j.ijnss.2025.12.009)
Supplement: Multimedia component 1 [file mmc1.docx]

**Appendix A**

Table S1. Strategies of literature search.

| Database | Retrieval strategies | | Number |
| --- | --- | --- | --- |
| PubMed | #1 | "Intubation, Intratracheal"[Mesh] | 4,324 |
|  | #2 | ("intratracheal intubat*") OR ("intratracheal tub*") OR ("endotracheal intubation*") OR ("intubations, intratracheal") OR ("intubation, indotracheal") OR ("intubations, endotracheal") OR ("endotracheal tube") OR ("not nasotracheal intubation") OR ("endotracheal tube intubation") OR ("ETT") OR ("trachea intubat*") OR ("tracheal cannula") OR ("trachea tub*") OR ("tracheal tub*") OR ("intracheal tube placement") OR ("tracheal tube insertion") OR ("tracheal intubat*") OR ("oral intubated patient") OR ("intubation, orotracheal") OR ("intubation, trachea") OR ("orotracheal intubat*") OR ("oral tracheal intubation") OR ("Intubation") OR ("windpipe tube") OR ("racheal cannula insertion") OR ("windpipe intubation") |  |
|  | #3 | "Respiration, Artificial"[Mesh] |  |
|  | #4 | ("artificial respiration*") OR ("artificial ventilation") OR ("artificial airway*") OR ("airway management") OR ("airway interventions") OR ("airway control") OR ("airway support") OR ("respirations, artificial") OR ("ventilation, mechanical") OR ("mechanical ventilation intubation") OR ("mechanical ventilat*") OR ("ventilation, artificial") OR ("ventilatory support") OR ("mechanical breathing support") OR ("ventilator-assisted breathing") OR ("mechanical respiratory support") OR ("positive pressure ventilation") |  |
|  | #5 | "Intensive care units" [Mesh] |  |
|  | #6 | ("critical care") OR ("ICU") OR ("medical intensive care unit") OR ("intensive-care settings") |  |
|  | #7 | ("tube fastener") OR ("tube-holder device") OR ("adhesive tape") OR ("cotton tape") OR ("twill tape") OR ("thomas tube holder") OR ("thomas fixation") OR ("elastic band") OR ("normal fixation adhesive tape") OR ("reinforced adhesive tape fixation") OR ("endotracheal tube") OR ("etts") OR ("airway holder") OR ("airway securing devices") OR ("secure airway") OR ("ET fixation methods") OR ("ett attachment device") OR ("tracheal tube fixing device ") OR ("catheter fixation device") OR ("fixation device") OR ("tracheal catheter fixation band") OR ("affixation device") OR ("teeth pad") OR ("endotracheal tube-holder device") OR ("bandage fixation") OR ("elastic adhesive strips") OR ("orotracheal intubation ixation methods") OR ("orotracheal intubation ixation methods") OR ("fixation tools") OR ("oral duct fixation method") OR ("sticking tape") OR ("gauze fixation") OR ("commercial cotton tube ties") OR ("holder") OR ("affixed") OR ("attach") OR ("fix*") OR ("regular") OR ("fasten*") OR ("stationary") OR ("stabilize") OR ("secure") OR ("set") OR ("immobil*") OR ("tighten") OR ("anchor") |  |
|  | #8 | #1 OR #2 |  |
|  | #9 | #3 OR #4 |  |
|  | #10 | #5 OR #6 |  |
|  | #11 | #7 AND #8 AND #9 AND #10 |  |
| Web of Science | #1 | TS=("intratracheal intubat*" OR "intratracheal tub*" OR "endotracheal intubation*" OR "intubations, intratracheal" OR "intubation, indotracheal" OR "intubations, endotracheal" OR "intubation, intratracheal" OR "endotracheal tube" OR "not nasotracheal intubation" OR "endotracheal tube intubation" OR " ETT" OR "trachea intubat*" OR " tracheal cannula" OR "trachea tub*" OR "tracheal tub*" OR "itracheal tube placement" OR "tracheal tube insertion" OR "tracheal intubat*" OR "oral intubated patient" OR "intubation, orotracheal" OR "intubation, trachea" OR "orotracheal intubat*" OR "oral tracheal intubation" OR "intubation" OR "windpipe tube" OR "racheal cannula insertion" OR "windpipe intubatton" OR "Intubation, Intratracheal") | 5,075 |
|  | #2 | TS=("respiration, artificial" OR "artificial respiration*" OR "artificial ventilation" OR "artificial airway*" OR "airway management" OR "airway interventions" OR "airway control" OR "endotracheal tube" OR "airway support" OR "respirations, artificial" OR "ventilation, mechanical" OR mechanical ventilation intubation" OR mechanical ventilat*" OR "ventilation, artificial" OR "mechanical ventilat*" OR "ventilation, artificial" OR "ventilatory support " OR "mechanical breathing support" OR "ventilator-assisted breathing" OR "mechanical respiratory support " OR "positive pressure ventilation") |  |
|  | #3 | TS=("Intensive care units" OR "Critical care" OR "ICU" OR "intensive-care settings" OR "Medical intensive care unit") |  |
|  | #4 | TS=("tube fastener" OR "tube-holder device" OR "adhesive tape" OR "cotton tape" OR "twill tape" OR "thomas tube holder" OR "thomas fixation" OR "elastic band" OR "normal fixation adhesive tape" OR "reinforced adhesive tape fixation" OR "endotracheal tube" OR "etts" OR "airway holder" OR "airway securing devices" OR "secure airway" OR "ET fixation methods" OR "ett attachment device" OR "tracheal tube fixing device" OR "catheter fixation device" OR "fixation device" OR "tracheal catheter fixation band" OR "affixation device" OR "teeth pad" OR "endotracheal tube-holder device" OR "bandage fixation" OR "elastic adhesive strips" OR "orotracheal intubation ixation methods" OR "orotracheal intubation ixation methods" OR "fixation tools" OR "oral duct fixation method" OR "sticking tape" OR "gauze fixation" OR "commercial cotton tube ties" OR "holder" OR "affixed" OR "attach" OR "fix*" OR "regular" OR "fasten*" OR "stationary" OR "stabilize" OR "secure" OR "set" OR "immobil*" OR "tighten" OR "anchor") |  |
|  | #5 | (#1 OR #2) AND ( #3 ) AND ( #4 ) |  |
| The Cochrane Library | #1 | MeSH descriptor: [Intensive Care Units] explode all trees | 639 |
|  | #2 | MeSH descriptor: [Intubation, Intratracheal] explode all trees |  |
|  | #3 | MeSH descriptor: [Respiration, Artificial] explode all trees |  |
|  | #4 | (Intensive Care Unit or ICU Intensive Care Units or Unit, Intensive Care or critically ill patients): ti, ab, kw (word variations have been searched) |  |
|  | #5 | (intratracheal intubat*): ti, ab, kw OR (intratracheal tub*): ti, ab, kw OR (endotracheal intubation): ti, ab, kw OR (intubations): ti, ab, kw OR (intratracheal intubat*): ti, ab, kw OR (intratracheal tub*): ti, ab, kw OR (endotracheal intubation): ti, ab, kw OR (intratracheal): ti, ab, kw OR (intubation, indotracheal): ti, ab, kw OR (intubations): ti, ab, kw OR (endotracheal): ti, ab, kw OR (intubation, intratracheal): ti, ab, kw OR (endotracheal tube): ti, ab, kw OR (not nasotracheal intubation): ti, ab, kw OR (endotracheal tube intubation): ti, ab, kw OR (ETT): ti, ab, kw OR (trachea intubat*): ti, ab, kw OR (tracheal cannula): ti, ab, kw OR (trachea tub): ti, ab, kw OR (tracheal tub*): ti, ab, kw OR (itracheal tube placement): ti, ab, kw OR (tracheal tube insertion): ti, ab, kw OR (tracheal intubat*): ti, ab, kw OR (intubated patient): ti, ab, kw OR (intubation, otracheal): ti, ab, kw OR (intubation, trachea): ti, ab, kw OR (otracheal intubat*): ti, ab, kw OR (tracheal intubation): ti, ab, kw OR (intubation): ti, ab, kw OR (windpipe tube): ti, ab, kw OR (racheal cannula insertion): ti, ab, kw OR (windpipe intubation): ti, ab, kw |  |
|  | #6 | (artificial respiration*): ti, ab, kw OR (artificial ventilation): ti, ab, kw OR (artificial airway*): ti, ab, kw OR (airway management): ti, ab, kw OR (airway interventions): ti, ab, kw OR (airway control): ti, ab, kw OR (airway support): ti, ab, kw OR (respirations, artificial): ti, ab, kw OR (ventilation, mechanical): ti, ab, kw OR (mechanical ventilation intubation): ti, ab, kw OR (mechanical ventilat*): ti, ab, kw OR (ventilation, artificial): ti, ab, kw OR (ventilatory support): ti, ab, kw OR (mechanical breathing support): ti, ab, kw OR (ventilator-assisted breathing): ti, ab, kw OR (mechanical respiratory support): ti, ab, kw OR (positive pressure ventilation): ti, ab, kw |  |
|  | #7 | (tube fastener): ti, ab, kw OR (tube-holder device): ti, ab, kw OR (adhesive tape): ti, ab, kw OR (cotton tape): ti, ab, kw OR (twill tape): ti, ab, kw OR (Thomas tube holder): ti, ab, kw OR (Thomas fixation): ti, ab, kw OR (elastic band): ti, ab, kw OR (normal fixation adhesive tape): ti, ab, kw OR (reinforced adhesive tape fixation): ti, ab, kw OR (endotracheal tube): ti, ab, kw OR (ETTs): ti, ab, kw OR (airway holder): ti, ab, kw OR (airway securing devices): ti, ab, kw OR (secure airway): ti, ab, kw OR (ET fixation methods): ti, ab, kw OR (ett attachment device): ti, ab, kw OR (tracheal tube fixing device): ti, ab, kw OR (catheter fixation device): ti, ab, kw OR (fixation device): ti, ab, kw OR (tracheal catheter fixation band): ti, ab, kw OR (affixation device): ti, ab, kw OR (teeth pad): ti,ab,kw OR (endotracheal tube-holder device):ti,ab,kw OR (bandage fixation):ti,ab,kw OR (elastic adhesive strips):ti,ab,kw OR (orotracheal intubation ixation methods):ti, ab,kw OR (orotracheal intubation fixation methods): ti, ab, kw OR (fixation tools): ti, ab, kw OR (oral duct fixation method): ti, ab, kw OR (sticking tape): ti, ab, kw OR (gauze fixation): ti, ab, kw OR (commercial cotton tube ties): ti, ab, kw OR (holder): ti, ab, kw OR (affixed): ti, ab, kw OR (attach): ti, ab, kw OR (fix*): ti, ab, kw OR (regular): ti, ab, kw OR (fasten*): ti, ab, kw OR (stationary): ti, ab, kw OR (stabilize): ti, ab, kw OR (secure): ti, ab, kw OR (set): ti, ab, kw OR (immobil*): ti, ab, kw OR (tighten): ti, ab, kw OR (anchor): ti, ab, kw |  |
|  | #8 | #1 or #4 |  |
|  | #9 | #2 or #5 |  |
|  | #10 | #3 or #6 |  |
|  | #11 | #7 and #8 and #9 and #10 |  |
| Embase | #1 | 'intratracheal intubat*':ab, ti OR 'intratracheal tub*': ab, ti OR 'endotracheal intubation*': ab, ti OR 'intubations, intratracheal': ab, ti OR 'intubation, indotracheal': ab, ti OR 'intubations, endotracheal': ab, ti OR 'intubation, intratracheal': ab, ti OR 'endotracheal tube': ab, ti OR 'not nasotracheal intubation': ab, ti OR 'endotracheal tube intubation': ab, ti OR 'ETT': ab, ti OR 'trachea intubat*': ab, ti OR 'tracheal cannula': ab, ti OR 'trachea tub*': ab, ti OR 'tracheal tub*': ab, ti OR 'itracheal tube placement': ab, ti OR 'tracheal tube insertion': ab, ti OR 'tracheal intubat*': ab, ti OR 'oral intubated patient': ab, ti OR 'intubation, orotracheal': ab, ti OR 'intubation, trachea': ab, ti OR 'orotracheal intubat*': ab,ti OR 'oral tracheal intubation':ab,ti OR 'intubation':ab,ti OR 'windpipe tube':ab,ti OR 'racheal cannula insertion':ab,ti OR 'windpipe intubatton':ab,ti OR 'intubation, intratracheal':ab,ti | 6,528 |
|  | #2 | 'intratracheal intubat*':ab, ti OR 'intratracheal tub*': ab, ti OR 'endotracheal intubation*': ab, ti OR 'intubations, intratracheal': ab, ti OR 'intubation, indotracheal': ab, ti OR 'intubations, endotracheal': ab, ti OR 'intubation, intratracheal': ab, ti OR 'endotracheal tube': ab, ti OR 'not nasotracheal intubation': ab, ti OR 'endotracheal tube intubation': ab, ti OR 'ETT': ab, ti OR 'trachea intubat*': ab, ti OR 'tracheal cannula': ab, ti OR 'trachea tub*': ab, ti OR 'tracheal tub*': ab, ti OR 'itracheal tube placement': ab, ti OR 'tracheal tube insertion': ab, ti OR 'tracheal intubat*': ab, ti OR 'oral intubated patient': ab, ti OR 'intubation, orotracheal': ab, ti OR 'intubation, trachea': ab, ti OR 'orotracheal intubat*': ab,ti OR 'oral tracheal intubation':ab,ti OR 'intubation':ab,ti OR 'windpipe tube':ab,ti OR 'racheal cannula insertion':ab,ti OR 'windpipe intubatton':ab,ti OR 'intubation, intratracheal':ab,ti |  |
|  | #3 | 'intensive care units ': ab, ti OR 'critical care ': ab, ti OR 'ICU ': ab, ti OR 'intensive-care settings ': ab, ti OR 'medical intensive care unit ': ab, ti |  |
|  | #4 | 'randomized controlled trial'/exp OR 'randomized controlled trial' OR 'randomized' OR 'placebo'/exp OR 'placebo' OR 'random' OR 'randomised' OR 'rct' |  |
|  | #5 | 'tube fastener' OR 'tube-holder device' OR 'adhesive tape'/exp OR 'adhesive tape' OR 'cotton tape' OR 'twill tape' OR 'thomas tube holder' OR 'thomas fixation' OR 'elastic band'/exp OR 'elastic band' OR 'normal fixation adhesive tape' OR 'reinforced adhesive tape fixation' OR 'endotracheal tube'/exp OR 'endotracheal tube' OR 'etts' OR 'airway holder' OR 'airway securing devices' OR 'secure airway' OR 'ett attachment device' OR 'tracheal tube fixing device' OR 'catheter fixation device' OR 'fixation device'/exp OR 'fixation device' OR 'oral duct fixation method' OR 'sticking tape' OR 'gauze fixation' OR 'commercial cotton tube ties' OR 'holder' OR 'affixed' OR 'attach' OR 'fix*' OR 'regular'/exp OR 'regular' OR 'fasten*' OR 'stationary' OR 'stabilize' OR 'secure' OR 'set' OR 'immobil*' OR 'tighten' OR 'anchor' |  |
|  | #6 | (#1 OR #2) AND ( #3 ) AND ( #4 ) AND ( #5 ) |  |
| Wanfang Databases | (主题:(重症监护病房) or 题名或关键词:(重症监护室 or 危重症患者 or 监护病房 or ICU ) )and (主题:(气管内插管) or 题名或关键词:(气管插管 or 气管插管术 or 气道内置管 or 辅助通气管道 or 呼吸机管道 or 机械通气 or 经口气管插管 or 口咽通气管 or 气管内导管))and (主题:(气管插管固定装置) or 题名或关键词:(口腔防护器 or 牙垫 or 改良牙垫 or 改良固定器 or 改良固定法 or 改良固定装置 or 口腔固定装置 or 固定装置 or 固定架 or 固定器 or 固定支架 or 固定带 or 固定方式 or 固定系统 or 固定组件 or 固定配件 or 锁定装置 or 固定带 or 外固定器 or 紧固带))) | | 177 |
| CNKI | SU= (气管内插管 + 气管插管 + 气管插管术 + 气道内置管 + 辅助通气管道 + 呼吸机管道 + 机械通气 + 经口气管插管 + 口咽通气管 + 气管内导管) AND SU=(口腔防护器 + 牙垫 + 改良牙垫 + 改良固定器 +改良固定法 + 改良固定装置 + 口腔固定装置 + 固定装置 + 固定架 + 固定器 + 固定支架 + 固定带 + 固定方式 + 固定系统 + 固定组件 + 固定配件 + 锁定装置 + 固定带 + 外固定器 + 紧固带 + 气管插管固定装置)AND SU=(ICU + 重症监护室 + 危重症患者 + 重症监护病房) | | 45 |
| CBM disc | ((((("重症监护病房"[常用字段] OR "重症监护室"[常用字段] OR "危重症患者"[常用字段] OR "监护病房"[常用字段] OR "ICU"[常用字段])) AND (("气管内插管"[常用字段] OR "气管插管"[常用字段] OR "气管插管术"[常用字段] OR "气道内置管"[常用字段] OR "辅助通气管道"[常用字段] OR "呼吸机管道"[常用字段] OR "机械通气"[常用字段] OR "经口气管插管"[常用字段] OR "气管内导管"[常用字段])))) AND (("气管插管固定装置"[常用字段] OR "固定器"[常用字段] OR "固定法"[常用字段] OR "固定装置"[常用字段] OR "固定方式"[常用字段] OR "固定装置"[常用字段] OR "固定带"[常用字段] OR "牙垫"[常用字段] OR "紧固带"[常用字段]))) | | 100 |
| Weipu Database | M= (气管内插管 + 气管插管 + 气管插管术 + 气道内置管 + 辅助通气管道 + 呼吸机管道 + 机械通气 +经口气管插管 + 口咽通气管 + 气管内导管) AND M=(口腔防护器 + 牙垫 + 改良牙垫 + 改良固定器 + 改良固定法 + 改良固定装置 + 口腔固定装置 + 固定装置 + 固定架 + 固定器 + 固定支架 + 固定带 + 固定方式 + 固定系统 + 固定组件 + 固定配件 + 锁定装置 + 固定带 + 外固定器 + 紧固带 + 气管插管固定装置) AND M=(ICU + 重症监护室 + 危重症患者 + 重症监护病房) | | 29 |

*Note*: CNKI = China National Knowledge Infrastructure; CBM disc = Chinese Biomedical Databases disc.

Table S2. The Details of each tracheal intubation fixation device.

| Types of each tracheal intubation fixation device | Code | Details |
| --- | --- | --- |
| Tracheal intubation fixator | A | Commercial endotracheal tube holder: The device usually consists of a "clamshell"-shaped silica colloid that wraps around and attaches onto the endotracheal tube via an adjustable, one-size-fits-all plastic clamp. The device plus ETT combination rests between the upper and the lower teeth, using the immobility of the maxilla for rigid fixation of the ETT to the patient |
| Dental pad fixation | B | Noncommercial endotracheal tube holding method; using dental pad (bite-block)and tying or fixing with adhesive tape or bandage, respectively, are more form-fitting to the patient's face |
| Adhesive tape fixation | C | Noncommercial method for securing endotracheal tubes, primarily utilizing adhesive materials composed of acrylic ester. The research focuses on three main types of adhesives, including 3M and Durapore™ |
| Bandage fixation | D | Noncommercial method for securing endotracheal tubes involves the use of a nonadhesive tape, primarily composed of a nylon/cotton blend or a pipeline tether strap |
| Hybrid fixation (adhesive tapes and straps for fixation) | E | Noncommercial endotracheal tube holding method; using adhesive tapes and bandage for fixation |

Table S3. Characteristics of the studies included in this network meta-analysis.

| Study (Author, Publication year, Country) | Sample size | | | Age (years) | | | Gender (%) | | | Primary diagnosis | Duration of mechanical ventilation (h) | | | APACHE II (score) | | Intervention | | Control | | Intervention duration | | Outcome measures |
| --- | --- | --- | --- | --- | --- | --- | --- | --- | --- | --- | --- | --- | --- | --- | --- | --- | --- | --- | --- | --- | --- | --- |
|  | IG | CG | | IG | CG | | Female | Male | |  | IG | CG | | IG | CG |  |  |  |  |  |  |  |
| Chen et al., 2017 [5], China | 45 | 45 | | 61.40 ± 9.10 | 61.00 ± 9.70 | | 16.7 | 83.3 | | COPD (56), Cancer (16), CAP (14), Bronchiectasis with infection (4) | 4.90 ± 0.70 | 5.00 ± 0.60 | | 16.60 ± 6.30 | 17.10 ± 5.70 | D | | C | | 16 months | | ①②③ |
| Niu et al., 2023 [12], China | 43 | 42 | | 58.42 ± 5.82 | 64.14 ± 6.39 | | 36.5 | 63.5 | | Respiratory failure (17), Pulmonary infection (13), Trauma (35), Cerebral infarction (11), Poisoning (9) | 5.70 ± 0.40 | 5.40 ± 0.30 | | / | / | D | | C | | 7 months | | ① |
| Genc and Yildiz,  2022 [13], Turkey | 30 | 30 | | / | / | | 43.3 | 56.7 | | Hypertension (24), COPD (1), Coronary artery disease (19), Cancer (2), Other (4), Not reported (10) | 1.15 ± 0.44 | | | / | / | D | | A | | 5 months | | ② |
| Landsperger et al., 2019 [30],  USA | 153 | 145 | | 53.20 ± 16.40 | 58.50 ± 16.10 | | 47.8 | 52.2 | | The most common is respiratory failure | 3.90 ± 3.40 | 3.90 ± 3.00 | | 27.40 ± 8.70 | 26.00 ± 8.90 | C | | A | | 12 months | | ①② |
| Zeng et al., 2016 [31], Singapore | 30 | 30 | | 63.50 | 62.00 | | / | / | | / | 3.20 ± 1.60 | 3.50 ± 1.80 | | / | / | C | | D | | 6 months | | ② |
| Bahadori et al.,  2022 [32], USA | 112 | 112 | | / | / | | 33.0 | 67.0 | | / | / | / | | / | / | C | | D | | 14 months | | ② |
| Xie, 2017 [33],  China | 40 | 40 | 40 | 43.98 ± 12.37 | 43.76 ± 15.70 | 43.76 ± 15.70 | 36.3 | 63.7 | 65.0 | / | 4.30 ± 1.30 | 4.30 ± 1.30 | 4.40 ± 1.20 | / | / | | C | A | E | | / | ①②③ |
| Hu et al., 2014 [34], China | 62 | 68 | | / | / | | 47.7 | 52.3 | | Orthopedic surgery (46), Craniocerebral injury (39), Infectious shock (18), Acute myocardial infarction (13), Respiratory failure (9), Poisoning (5) | 2.20 ± 0.70 | | | / | / | A | | B | | 9 months | | ①②③ |
| Chen et al., 2016 [35], China | 18 | 18 | | / | / | | 50.0 | 50.0 | | Cardiovascular disease (17), Cancer (13), Trauma (6) | 6.00 ± 0.80 | | | / | / | A | | D | | 15 months | | ①② |
| Qian and Lu, 2022 [36], China | 116 | 116 | | 63.25 ± 2.60 | 61.81 ± 3.40 | | 31.0 | 69.0 | | / | / | / | | / | / | A | | B | | 7 months | | ①②③ |
| Zhu et al., 2020 [37], China | 53 | 54 | | / | / | | 43.9 | 56.1 | | Respiratory failure (10), Severe pneumonia (6), ARDS (19), Craniocerebral injury (18), Cerebral hemorrhage (8), Stroke (6), Gastrointestinal bleeding (4), Digestive tract tumors (2), Severe pancreatitis (4), Abdominal infection (18), Renal failure (7), Trauma (5) | / | / | | / | / | C | | B | | 4 months | | ①②③ |
| Wang et al., 2013 [38], China | 30 | 32 | | / | / | | 41.9 | 58.1 | | Severe pancreatitis (6), Infectious shock (8), Respiratory failure (24), Myocardial infarction (10), Trauma (12), Other (2) | 6.00 ± 2.60 | | | / | / | E | | B | | 10 months | | ①②③ |
| Ma and Shen, 2014 [39], China | 54 | 54 | | 61.35 ± 9.06 | 60.98 ± 9.87 | | 20.4 | 79.6 | | Respiratory failure (43) , Poisoning (34), Cerebral infarction (18), Other (13) | 5.00 ± 0.60 | | | 16.00± 1.80 | 14.70 ± 2.10 | D | | B | | 25 months | | ① |
| Miu and Tang, 2016 [40], China | 54 | 54 | | / | / | | 43.5 | 56.5 | | / | / | / | | / | / | C | | B | | / | | ② |
| Hu et al., 2009 [41],  China | 39 | 37 | | 51.70 ± 16.70 | 53.70 ± 15.30 | | 25.0 | 75.0 | | Poisoning (31), Respiratory failure (17), Trauma (14), Other (14) | / | / | | / | / | E | | B | | 27 months | | ③ |
| Liu and Lu, 2022 [42],  China | 40 | 40 | | / | / | | 40.0 | 60.0 | | Cerebral hemorrhage (34), Hemorrhagic shock (26), Respiratory failure (20) | / | / | | / | / | D | | B | | 9 months | | ①②③ |

*Note:* IG = Intervention group, CG = Comparator group; COPD = Chronic obstructive pulmonary disease; CAP = Community-acquired pneumonia; ARDS = Acute respiratory distress syndrome; APACHE II = Acute Physiology and Chronic Health Evaluation II; A = Tracheal intubation fixator; B = Control (Dental pad fixation); C = Adhesive tape fixation; D = Bandage fixation; E = Hybrid fixation; ① = Catheter displacement; ② = Facial pressure injury: Pressure Injury Staging System(NPUAP); ③ = Pain; NPUAP = National Pressure Ulcer Advisory Panel; VAS = visual analogue scale

Table S4. Nodal analysis of catheter displacement.

| Intervention | *P* | Direct | Indirect | Network |
| --- | --- | --- | --- | --- |
| C/A | 0.093 | 1.70 | 0.06 | 0.45 |
| A/B | 0.904 | 0.20 | 0.26 | 0.22 |
| D/C | **0.040** | 22.0 | 0.29 | 2.70 |
| A/D | 0.060 | 0.05 | 4.70 | 1.20 |
| C/B | 0.800 | 2.90 | 1.60 | 2.00 |
| D/B | 0.722 | 4.00 | 8.90 | 5.40 |

Table S5. Nodal analysis of facial pressure injury.

| Intervention | *P* | Direct | Indirect | Network |
| --- | --- | --- | --- | --- |
| C/A | 0.095 | 1.70 | 0.06 | 0.45 |
| A/B | 0.873 | 0.05 | 0.02 | 0.04 |
| D/C | **0.040** | 22.00 | 0.29 | 2.70 |
| A/D | 0.062 | 0.05 | 4.60 | 1.20 |
| C/B | 0.807 | 2.80 | 1.60 | 2.00 |
| D/B | 0.718 | 4.00 | 9.10 | 5.40 |

Table S6. Nodal analysis of pain.

| Intervention | *P* | Direct | Indirect | Network |
| --- | --- | --- | --- | --- |
| C/A | 0.538 | 1.20 | 0.07 | 0.31 |
| A/B | 0.873 | 0.05 | 0.02 | 0.04 |
| D/C | 0.236 | 66.00 | 0.34 | 9.10 |
| A/D | 0.434 | 0.49 | 19.0 | 2.80 |
| C/B | 0.521 | 50.00 | 1.80 | 7.90 |
| D/B | 0.604 | 11.00 | 2.30 | 74.0 |

Table S7. The heterogeneity test results of various intubation fixation devices.

| Intervention | catheter displacement | | facial pressure injury | | pain | |
| --- | --- | --- | --- | --- | --- | --- |
|  | *P* | *I^2^*(%) | *P* | *I^2^*(%) | *P* | *I^2^*(%) |
| C/A | 0.530 | 96.0 | 0.640 | 81.0 | 0.110 | / |
| A/B | 0.000 | 93.0 | 0.440 | 98.0 | <0.001 | 40.0 |
| E/B | 0.910 | / | 0.020 | / | <0.001 | 0 |
| D/C | 0.000 | 11.0 | 0.720 | 38.0 | / | / |
| A/D | 0.020 | / | 0.600 | 92.0 | / | / |
| C/B | 0.020 | / | 0.370 | 86.0 | 0.770 | / |
| D/B | 0.390 | 71.0 | 0.050 | / | 0.180 | / |

*Note:* A = Tracheal intubation fixator; B = Control (Dental pad fixation); C = Adhesive tape fixation; D = Bandage fixation; E = Hybrid fixation.
