# Supplementary material for: Comparative effectiveness of various intubation fixation devices for patients undergoing tracheal intubation in the ICU: A systematic review and network meta-analysis
Source: Int J Nurs Sci. 2025 Dec 17;13(3):340–7. doi: 10.1016/j.ijnss.2025.12.009 (PMC13245521; doi:10.1016/j.ijnss.2025.12.009)
Supplement: Multimedia component 2 [file mmc2.docx]

**Identification of studies via databases and registers**

**Identification of studies via other methods**

**Identification**

Record identified through database searching (*n* = 16,917): PubMed (*n* = 4,324); Embase (*n* = 6,528); Web of Science (*n* = 5,075); the Cochrane Library (*n* = 639); CNKI (*n* = 45); Wanfang Databases (*n* = 177); CBM disc (*n* = 100); Weipu Database (*n* = 29)

Records identified from: Citation searching

(*n* = 6)

Records removed before screening:

Duplicate records removed

(*n* = 6,909)

Records excluded after review of the title and abstract (*n* = 9,671):

Not an RCT (*n* = 2,809)

Irrelevant population (*n* = 3,200)

Intervention mismatch (*n* = 1,531)

Animal studies (*n* = 1,274)

Non-English/Chinese literature (*n* = 486)

Duplicate publications (*n* = 371)

在标题/摘要审查后排除的记录（n=9671）：

• 不相关人群（n=3200）

• 非干预性研究（n=2800）

• 干预措施不匹配（n=1500）

• 动物研究（n=1200）

• 非英语/中文文献（n=600）

• 重复发表（n=371）

**Screening**

Records screened

(*n* = 10,008)

Reports sought for retrieval

(*n* = 6)

Reports not retrieved

(*n* = 3)

Reports sought for retrieval

(*n* = 337)

Reports not retrieved (*n* = 12)

Reports excluded:

Ineligible outcomes (*n* = 1)

Not an RCT (*n* = 1)

Reports excluded (*n* = 310):

Not an RCT (*n* = 118)

Irrelevant population (*n* = 31)

Intervention mismatch (*n* = 19)

Ineligible outcomes (*n* = 60)

Non-peer-reviewed sources (*n* = 27)

Protocol/ongoing studies (*n* = 29)

Duplicate publications (*n* = 26)

Reports assessed for eligibility

(*n* = 3)

Reports assessed for eligibility

(*n* = 325)

Studies included in network meta-analysis (*n* = 16)

**Included**

Figure S1. Flow diagram for the search for and selection of the included studies. *Note:* CNKI = China National Knowledge Infrastructure; CBM disc = Chinese Biomedical Databases disc.

Figure S2. The risk of bias assessment of the included research.

| 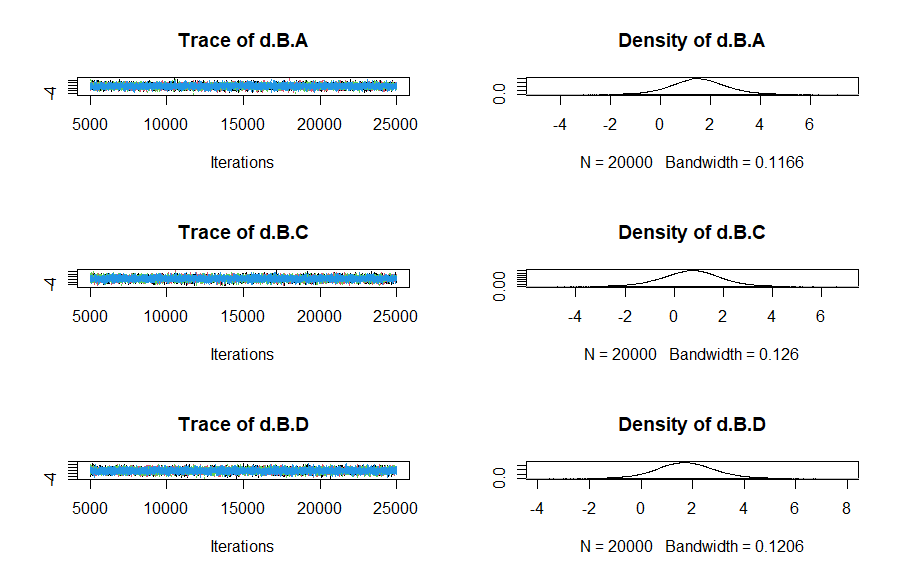 | 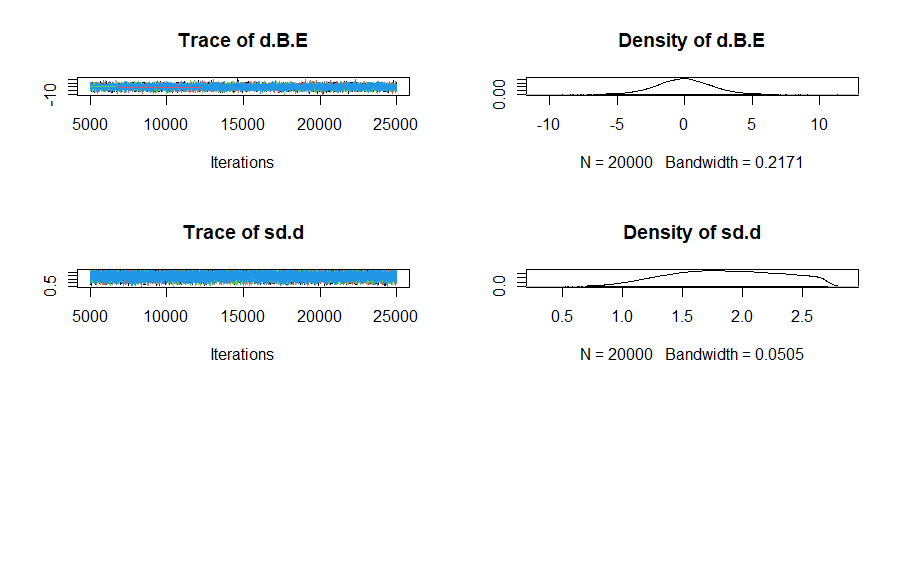 |
| --- | --- |

Figure S3. The Potential Scale Reduction Factor (PSRF) values pertaining to catheter displacement.


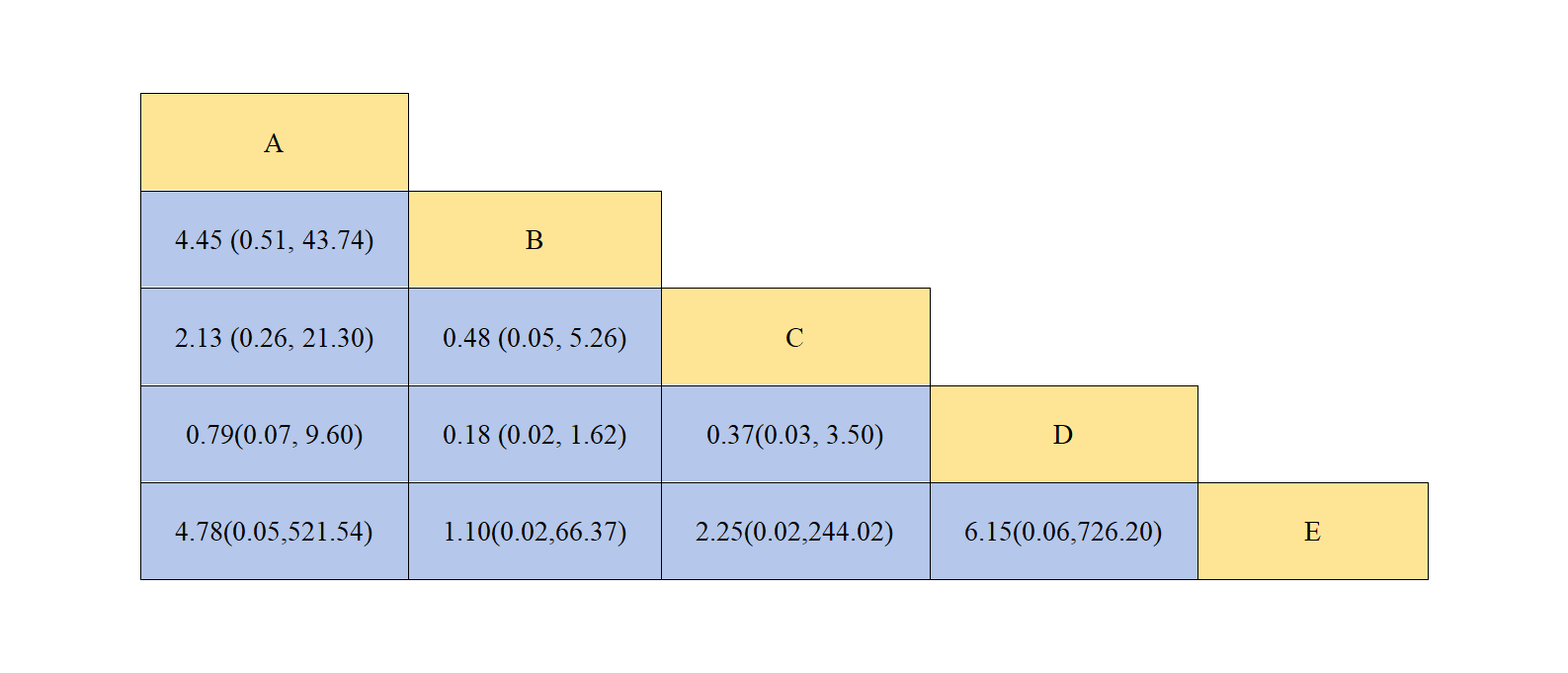


Figure S4. Relative effects of different devices for catheter displacement.

| 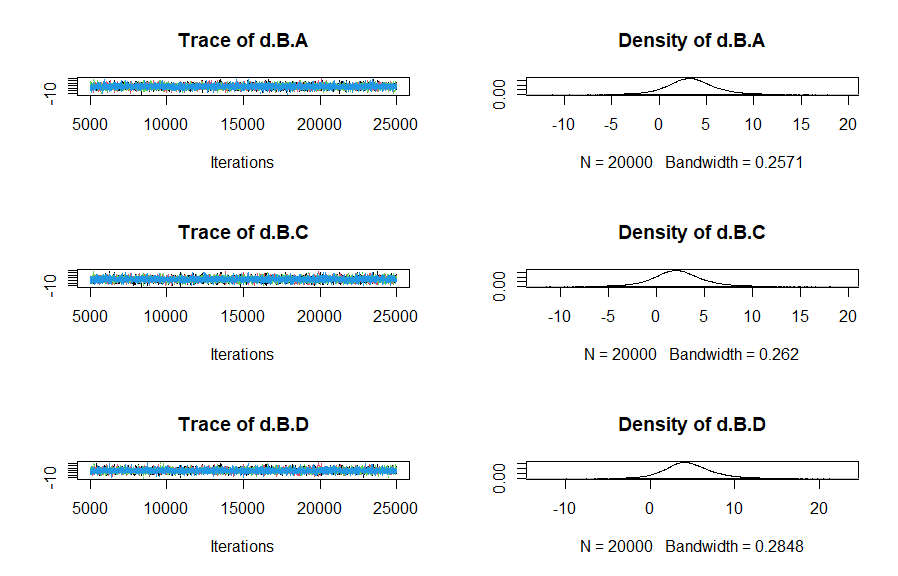 | 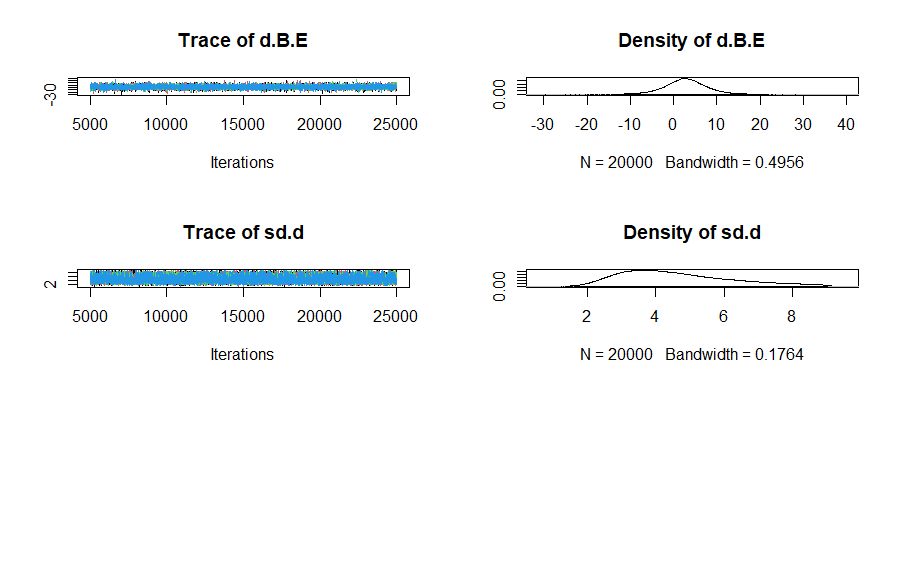 |
| --- | --- |

Figure S5. The Potential Scale Reduction Factor (PSRF) values pertaining to facial pressure injury.


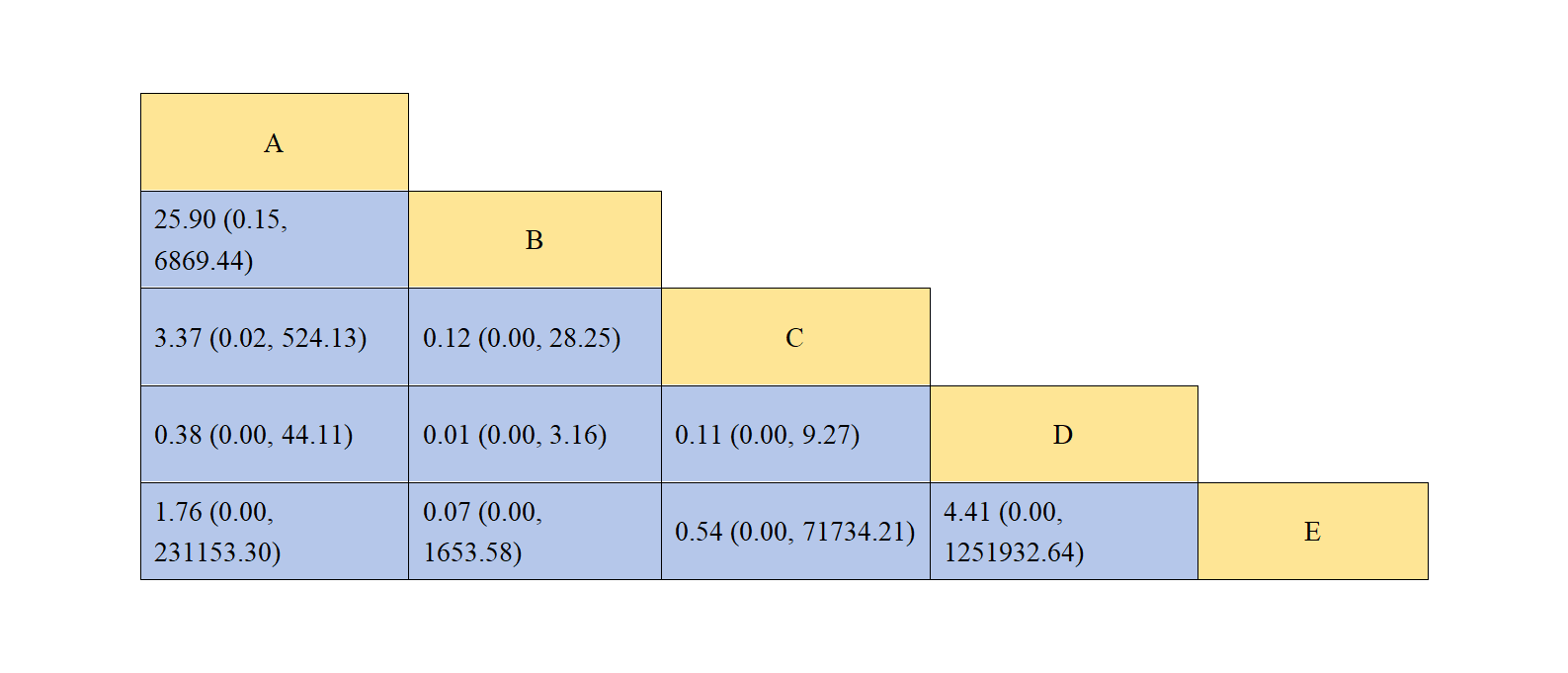


Figure S6. Relative effects of different devices for facial pressure injury

| **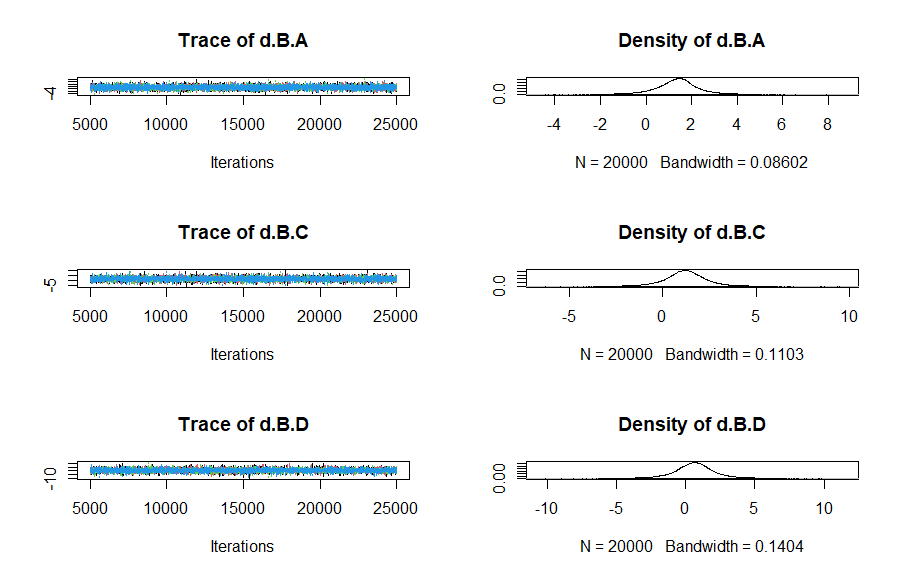** | **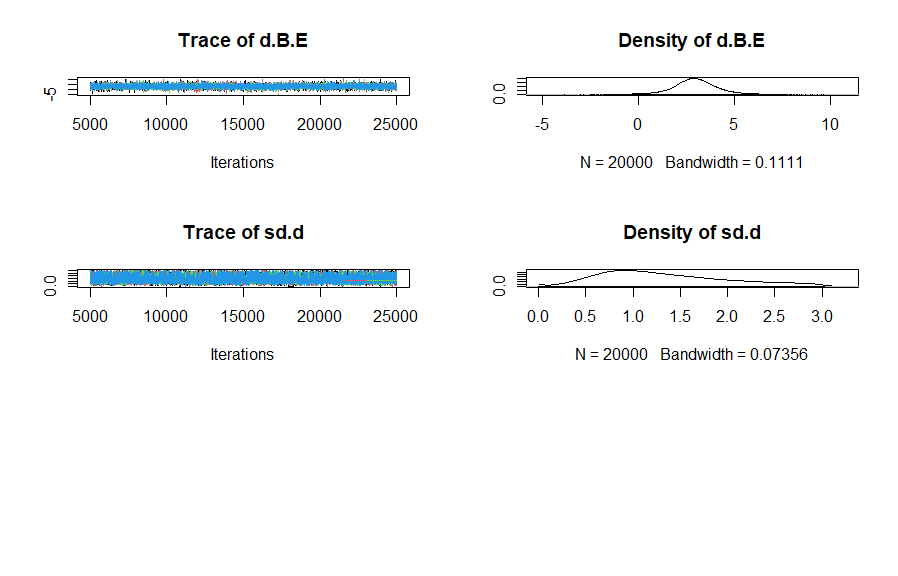** |
| --- | --- |

Figure S7. The Potential Scale Reduction Factor (PSRF) values pertaining to pain.

**
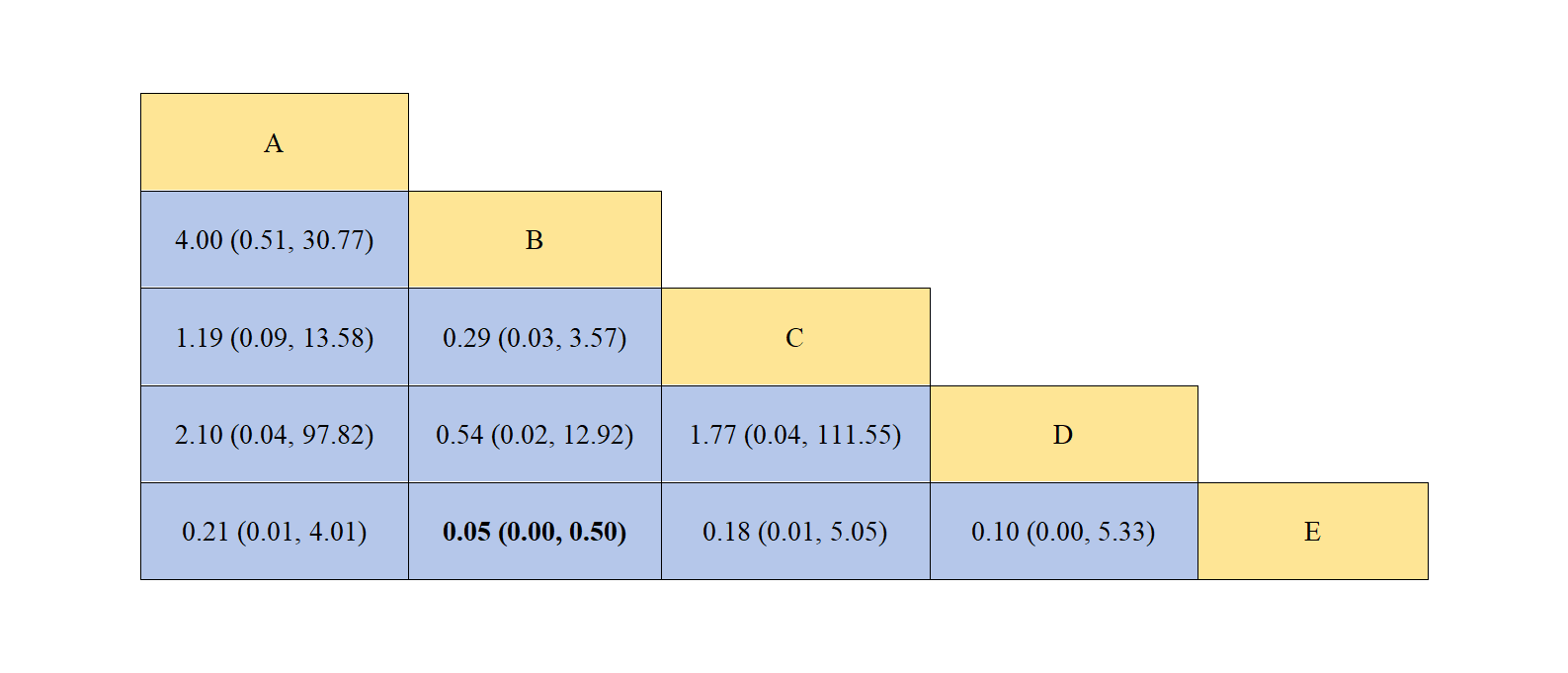
**

Figure S8. Relative effects of different devices for pain.

**
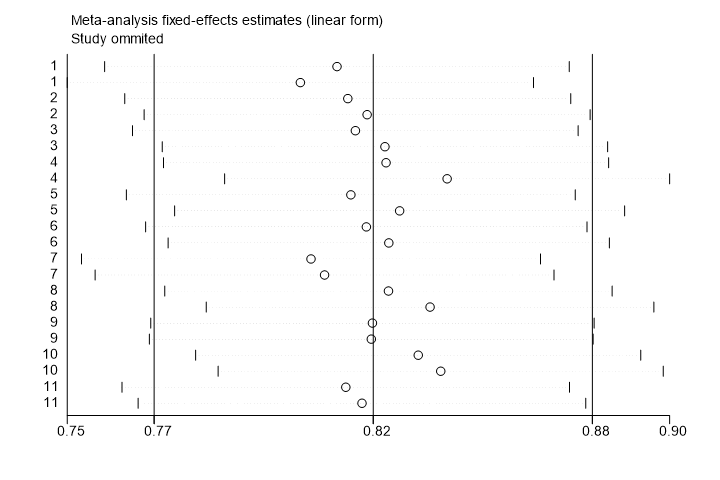
**

Figure S9. Sensitivity analysis of catheter displacement.


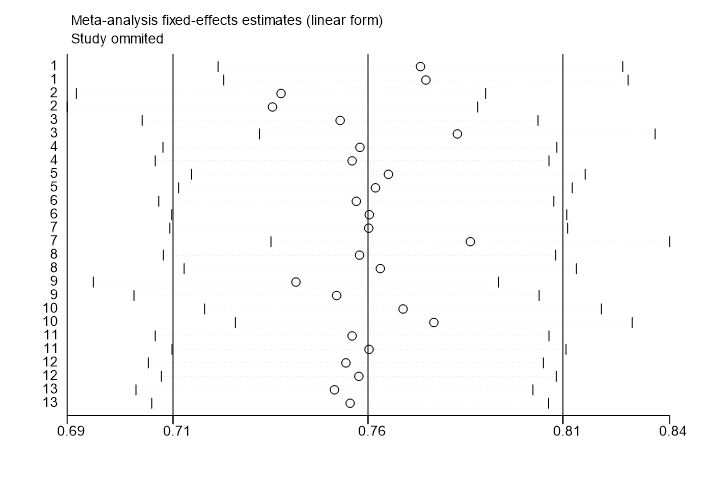


Figure S10. Sensitivity analysis of facial pressure injury.

**
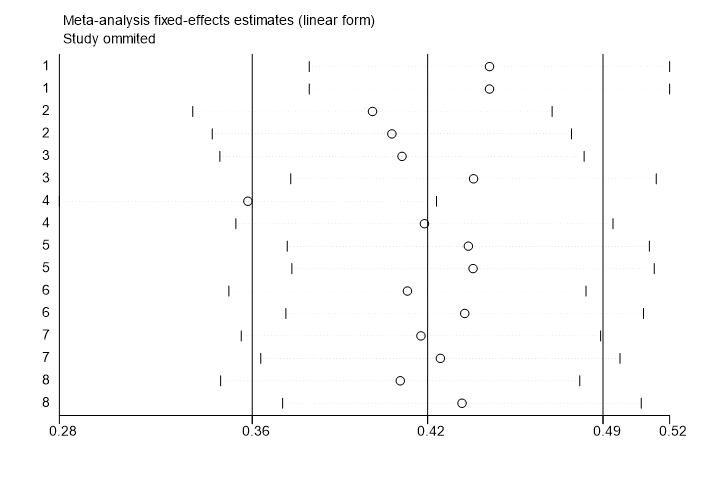
**

Figure S11. Sensitivity analysis of pain.


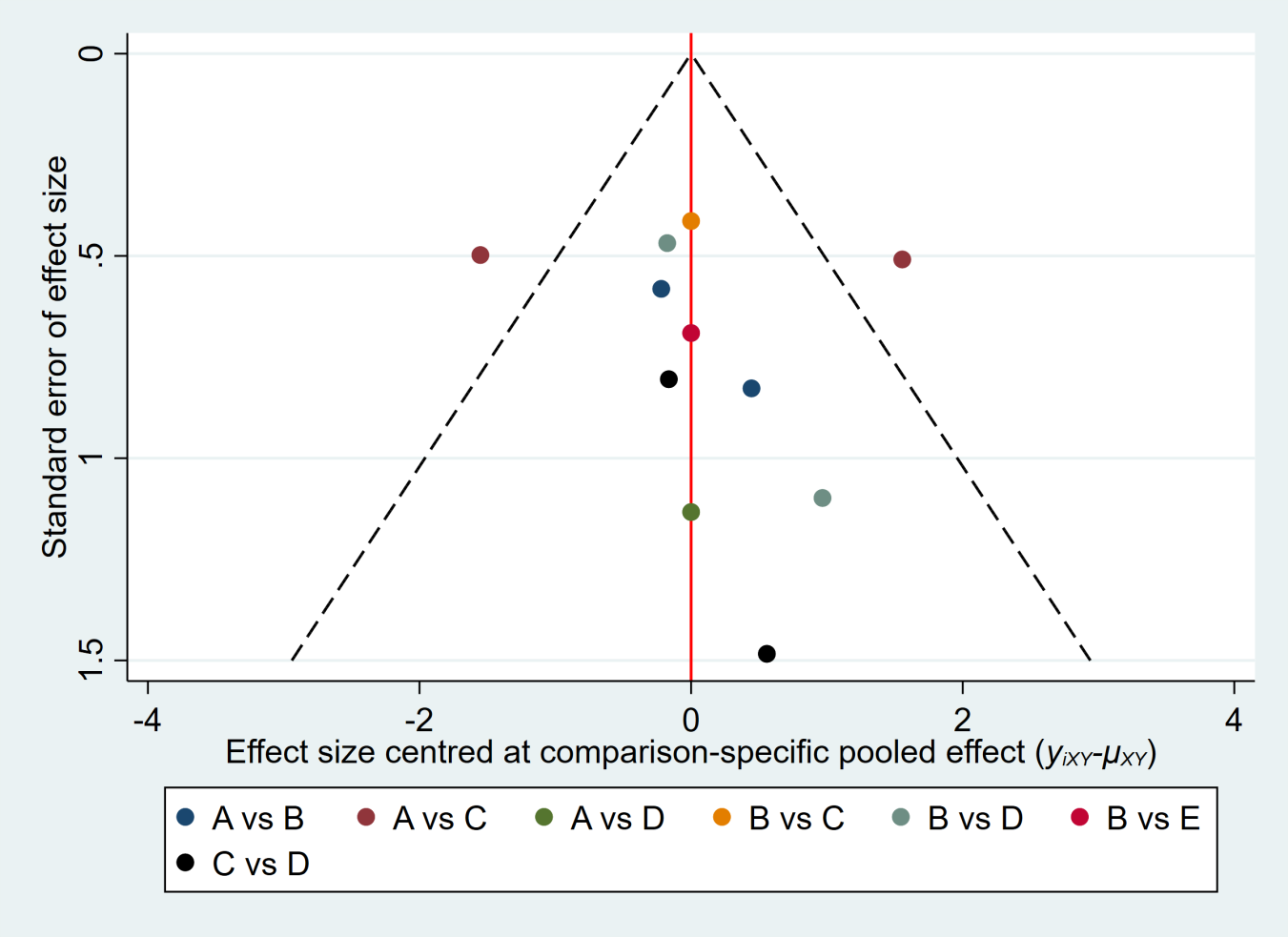


Figure S12. The publication bias funnel plot of catheter displacement.


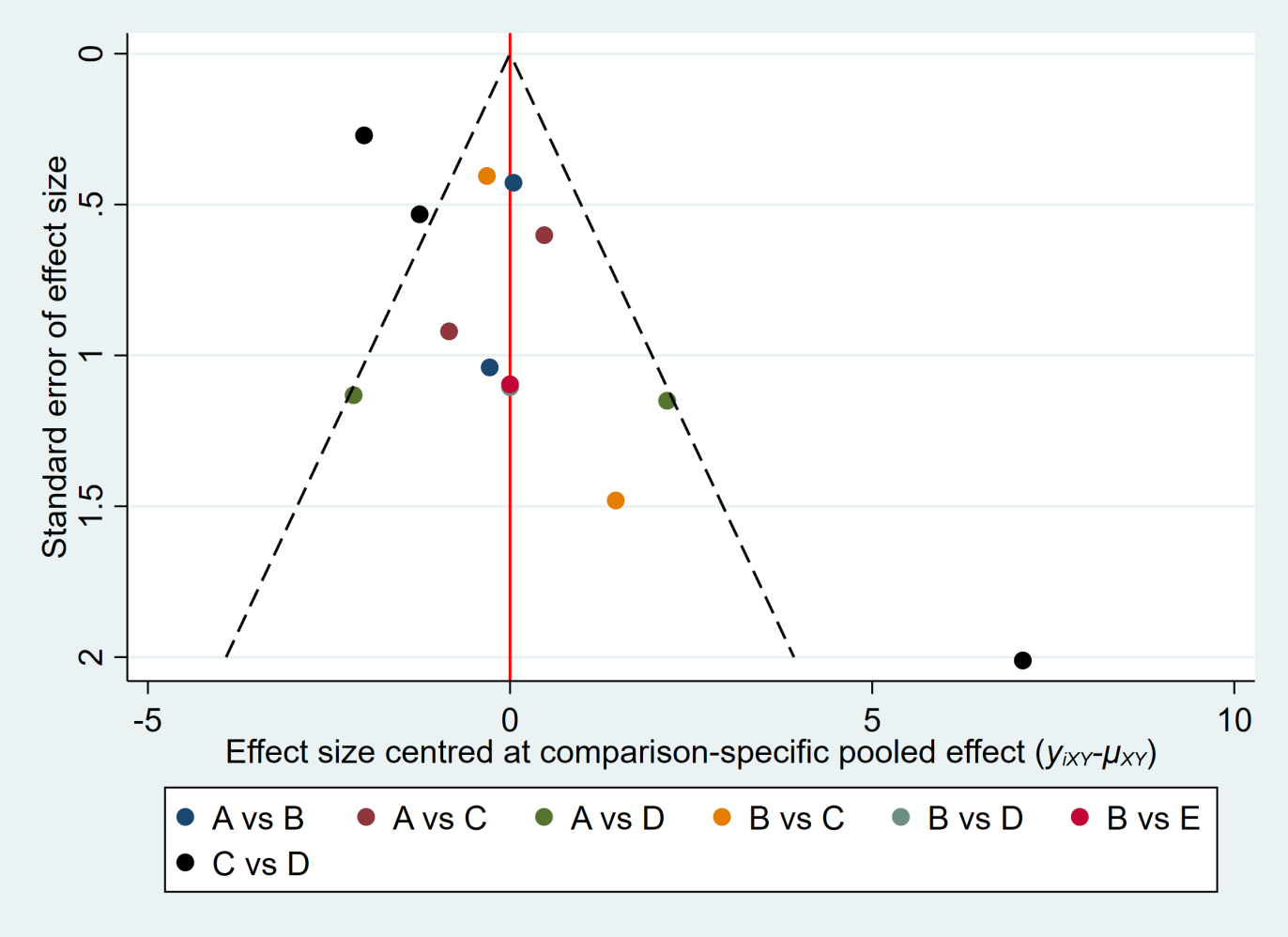


Figure 13. The publication bias funnel plot of facial pressure injury.

*Note:* A = Tracheal intubation fixator; B = Control (Dental pad fixation); C = Adhesive tape fixation; D = Bandage fixation; E = Hybrid fixation.
