# Supplementary material for: Comparative effectiveness of various intubation fixation devices for patients undergoing tracheal intubation in the ICU: A systematic review and network meta-analysis
Source: Int J Nurs Sci. 2025 Dec 17;13(3):340–7. doi: 10.1016/j.ijnss.2025.12.009 (PMC13245521; doi:10.1016/j.ijnss.2025.12.009)
Supplement: Multimedia component 3 [file mmc3.docx]

**不同气管插管固定装置在ICU患者中应用效果的比较：一项系统评价与网状Meta分析**

黄泽曦，崔念奇，郑艳敏，杨涧沿，平月丽，沙瑞芹，刘永刚，田莹

【**摘要**】

**目的** 该研究比较了并排序重症监护室中用于经口气管插管患者的不同插管固定装置的有效性。

**方法** 系统检索PubMed、the Cochrane Library、Web of Science、Embase、中国知网、万方、维普数据库及中国生物医学文献数据库，检索时限自建库至2025年3月。纳入比较不同气管插管固定装置在ICU患者中应用的随机对照试验，以导管移位、面部压力性损伤及疼痛为主要结局指标，采用网状Meta分析整合直接与间接证据，计算各装置的比值比及累积排序概率。

**结果** 共纳入16项随机对照试验，涉及5种气管插管固定装置。网状Meta分析结果显示，在导管移位和面部压力性损伤方面，各装置间差异均无统计学意义；但在缓解疼痛方面，混合固定法（联合使用粘胶敷料与固定带）显著优于牙垫固定（*OR*=16.27，95%*CI*：3.56~74.30），且在减轻疼痛方面的排序概率中最高（78%）。

**结论** 目前尚无一种固定装置在所有结局指标上均表现最优，但混合固定法在降低患者疼痛方面具有明显优势。未来仍需开展更多高质量、大样本的随机对照试验，以进一步验证其在不同临床情境下的适用性及其他结局指标的效能。

【**关键词】** 导管位移；气管插管；重症监护室；固定装置；网状Meta分析

**通信作者：**田莹，E-mail：[tianyychen@163.com](mailto:tianyychen@163.com)
